# No Routine Control Measurements of C-Reactive Protein in Uneventful Postoperative Evolution After Debridement for Infected (Diabetic) Foot Surgery

**DOI:** 10.3390/jcm14124122

**Published:** 2025-06-11

**Authors:** Jonas Liebe, Laura Soldevila-Boixader, İnci Yιldιz, Pascal R. Furrer, Peter Jans, Arnd Viehöfer, Stephan Wirth, İlker Uckay

**Affiliations:** 1 Foot Surgery, Department of Orthopedic Surgery, Balgrist University Hospital, Forchstrasse 340, 8008 Zurich, Switzerland; liebejonas1993@gmail.com (J.L.); inci.yildiz@balgrist.ch (İ.Y.); pascal.furrer@balgrist.ch (P.R.F.); arnd.viehoefer@balgrist.ch (A.V.); stephan.wirth@balgrist.ch (S.W.); 2 Infectious Disease Service, IDIBELL-Hospital Universitari Bellvitge, Universitat de Barcelona, Feixa Llarga s/n, Hospitalet de Llobregat, 08907 Barcelona, Spain; lsoldevilab@csi.cat; 3 Infectiology, Unit for Clinical and Applied Research, Balgrist University Hospital, 8008 Zurich, Switzerland; 4 Medical Informatics Service, Balgrist University Hospital, 8008 Zurich, Switzerland; peter.jans@balgrist.ch

**Keywords:** surgical-site infection, diabetic orthopedic foot surgery, CRP, postoperative follow-up, treatment failures, associations, predictive accuracy

## Abstract

**Background/Objectives:** In orthopedic (diabetic) foot surgery, the serum C-reactive protein (CRP) level is frequently measured not only as a diagnostic tool, but also as a control inflammatory marker in the follow-up of postoperative surgical-site infections (SSIs) **Methods**: We investigated the predictive value of the post-debridement routine (control) serum CRP level in adult (diabetic) patients with an SSI in the foot. We excluded community-acquired (diabetic foot) infections and focused on the predictive accuracy of routine (control) CRP measurements in terms of ultimate therapeutic failures. **Results:** The median pre- and postoperative CRP levels were 25 mg/L and 8.8 mg/L, respectively. In group comparisons and multivariate assessment, neither the immediate (relative and absolute) drop in the serum CRP level, nor its values between 5 and 8 weeks and between 11 and 14 weeks predicted the failure risk of 19%. In contrast, in cases of surprisingly elevated CRP levels, this finding leads to unnecessary radiological (median costs approximatively USD 200), clinical, microbiological urinary sample (median costs USD 50), and laboratory (one CRP sample USD 10) exams. These additional exams also likely prolong the duration of hospitalization by one to two days (e.g., whilst awaiting the microbiological results) and often generate unnecessary consultations among internist and/or infectious diseases experts (USD 50). **Conclusions**: Routine, postoperative CRP monitoring during the treatment of established orthopedic (diabetic) foot SSIs is unnecessarily costly, and should be avoided in favor of clinical surveillance of the postoperative evolution.

## 1. Introduction

Surgical-site infections (SSIs) following elective orthopedic foot and ankle surgeries are frequent complications with an incidence ranging from 1.9% up to 4.2% [1,2,3,4,5], or 9.4%, respectively [2], including for patients with concomitant diabetes mellitus [1]. Serum inflammatory markers such as C-reactive protein (CRP) are widely used as a supplementary tool in the therapeutic follow-up of infected patients, alongside clinical diagnosis and microbiological (and histological) confirmation of infection, although no clinical thresholds exist on that matter. The infectious diseases experts emphasize that no therapeutic decision depends on the serum CRP level per se. The reasons for this surgical tradition may be to monitor for good evolution, to estimate a prognosis, or to anticipate problems. The scientific benefit remains unclear, since the clinical evaluation provides all of the information needed, and the CRP peak always lags two days behind the triggering event [6,7].

Undoubtedly, postoperative CRP levels can correlate with new problems such as progredient ischemia, fracture, or thrombosis, but the CRP level does not really help whenever these problems are recognized visually. On the contrary, in cases of a surprisingly elevated CRP level, the responsible clinicians may perform unnecessary radiological and laboratory exams (X-rays or even magnetic resonance imaging (MRI), superficial microbiological wound swabs, urinary cultures), all of which ultimately increase the cost or lead to precautional antibiotic use without benefits for the patients.

Several studies have highlighted the limitations of routine (control) and iterative CRP monitoring in patients undergoing orthopedic surgery [8,9,10]. Specifically, regarding ischemic and implant-free community-acquired diabetic foot infections (DFIs), Pham et al. denied the utility of such routine CRP measurement during antibiotic therapy for DFIs, as it fails to predict treatment failures [9]. Similarly, Furrer et al. found no benefits of other routine serum laboratory controls (leukocytosis, platelets, and CRP) in the practical management of postoperative, community-acquired DFIs [10]. In both studies, the CRP level reflected both the infection and a large part of the non-infectious postoperative inflammation. Critics might argue that in both previous studies [9,10], the clinical infections were community-acquired, chronic infections that were complicated by concomitant ischemia and pre-existing ulcerations, which might have interfered with the CRP levels. In contrast to these prior publications, we want to evaluate the routine CRP controls in the aftermath of acute orthopedic foot SSIs, mostly in the hospitalization setting, without the interference of (chronic) ischemia and other concomitant reasons for non-infectious skin breakdowns.

In this study, we evaluated the widespread practice of CRP sampling in adult orthopedic patients with an SSI following (non-ischemic) diabetic orthopedic foot surgery. Importantly, we did not aim to associate a high postoperative CRP level with various complications such as thrombosis, ischemia, new infections, mortality, or fractures, for which a broader range of literature is available [11]. By focusing on acute SSIs, we limit the CRP-related reasons to the infectious nature of SSIs and question the utility of routine CRP monitoring in anticipating therapeutic failures. We believe that our findings could significantly impact clinical practice by reducing futile testing and (antibiotic) costs, without compromising outcomes.

## 2. Materials and Methods

We retrospectively analyzed all SSIs after elective (diabetic) foot surgery in our tertiary (diabetic) foot center at the Balgrist University Hospital in Zurich, Switzerland, from January 2014 to August 2022 [1]. We used data mining from the hospital’s own medical databases and confirmed the presence of SSIs by evaluating the electronic files. We included all SSIs (first episode) following adult elective foot and ankle surgeries in our patients that were older than 18 years with a minimum surveillance of two years after the start of therapy, and with detailed information available prior to database closure (31 August 2024).

### 2.1. Study Definitions, Costs, and Criteria

The definition of an SSI is based on international criteria [1,2,3,4,12]. SSIs are acute infections that usually occur at the surgical operating site within 30 days after the index foot surgery. They are believed to be acquired intraoperatively. In contrast, community-acquired foot infections are caused by other events and underlying co-morbidities of frail patients (e.g., minor trauma, polyneuropathy, ischemic skin breakdown) without undergoing recent surgery. The distinctions based on the patient’s history and medical records are widely accepted as such in the scientific literature.

We defined “remission” as the absence of any clinical, laboratory, or radiological data indicating suspicion of a persistence or recurrence of infection at the same anatomical localization after a minimal follow-up time of six months. Inversely, we defined “failure” as any indication for an unplanned surgical revision and/or antibiotic therapy. An uneventful evolution revealed no major new problems, such as postsurgical fractures, pneumonia, heart attacks, or pulmonary embolisms, which shifted the therapeutic course towards this new problem. We defined “ischemia “and “polyneuropathy” on clinical grounds. Both are hallmarks of diabetic foot problems. Specifically, for this study, we considered the foot to be ischemic or neuropathic if there was a clinical intervention for either indication (e.g., revascularization or targeted therapy).

The exclusion criteria were described in previous studi [7,9,10] and are briefly defined as external patients that we treated only partially or patients with recurrent infections; amputations without residual infection; infection episodes with prior emergency index surgery; open fractures; foot infections extending beyond the ankle (e.g., gas gangrene, necrotizing fasciitis and other rapidly spreading severe soft-tissue infections); insufficient documentation; atypical pathogens such as *Actinomyces* spp., fungi, or mycobacteria; concomitant severe and remote infections such as endocarditis or brain abscesses; and, most importantly, the presence of a community-acquired DFI with and without ischemia, infected foot ulcers, and/or severe neuropathy. In contrast, a patient with well-regulated concomitant diabetes mellitus was allowed to be included in the study if he/she had developed a non-ischemic SSI [1]. We equally excluded all cases with any postoperative complication during the SSI. This large exclusion list avoided the presence of community-acquired, chronic, ulcer-related or ischemic diabetic foot infections that are a different clinical entity. Postoperatively, the CRP measurement and its timing were at the discretion of the treating surgeons. One CRP sample costs approximatively CHF 10, equaling USD 10. A CRP value of <10 mg/L was considered to be normal according to our laboratory (ZLZ; Zentral Labor Zürich). We avoided intraarticular CRP measurements.

Other hidden costs associated with routine CRP sampling (especially when levels are surprisingly elevated) are radiological (estimated median costs approximatively USD 200), clinical, microbiological urinary sample (median costs USD 50), and laboratory (one CRP sample costs USD 10) exams. These additional exams also likely prolong the duration of hospitalization by one to two days (e.g., when awaiting the microbiological results) and often generate unnecessary consultations among internist and/or infectious diseases experts (USD 50).

### 2.2. Statistical Analyses

The primary outcome was remission, and/or inversely, failure, after the end of treatment for an SSI after orthopedic (diabetic) foot surgery. The secondary outcomes were dynamic changes in the CRP level during therapy. For the group comparisons, we used the Pearson’s χ^2^ or the Wilcoxon rank sum test. For the large case mix and to compare the different CRP values within the entire inhomogeneous study population, we performed a multivariate unconditional logistic regression analysis with the outcome “failure”. We included 5–8 predicter variables per number of outcome variables. As this was a side study of prior publications, we had no formal sample size requirements. We used STATA^™^ software (Version 15.0; College Station, TX, USA); *p*-values of <0.05 (two-tailed) were considered significant.

## 3. Results

### 3.1. Study Population

We retrospectively analyzed a cohort of foot surgeries that were carried out between 1 January 2014 and 31 August 2022 [1]. Overall, we assessed 6138 elective surgeries with an overall SSI risk of 1.88%. The reasons for elective surgeries were multiple, ranging from hallux operations to arthrodesis, reconstructions and total ankle joint prostheses, with or without hardware. The spectrum of operative techniques and indications corresponded to the usual patient population of a tertiary center. For the final analysis, we included 36 SSI cases in 36 different adult foot patients with complete data. The median age was 60 years (interquartile range (IQR, 48–77 y), and fifteen patients were female (42%). The median body mass index was 28.7 kg/m^2^ (IQR, 24–33) and the number of co-morbidities was high (diabetes n = 5, sarcoidosis n = 1, rheumatic arthritis n = 1). Half of the patients (18/36; 50%) yielded an American Society of Anesthesiologists’ Score of over 2 points and five (14%) had a superinfected Charcot foot arthropathy. The median length of hospital stay in acute care surgery was 12 days (IQR, 7–19 d).

### 3.2. Surgical-Site Infections, Pathogens, Therapies, and Outcomes

We detected sixteen different microbiological SSI constellations, of which one third were polymicrobial (12/36; 33%). The both most frequent mono-infections were due to *Staphylococcus aureus* (17%) and *S. epidermidis* (6%). Two-thirds (67%) of the SSIs involved bone, and in seven cases (19%), the infected (or contaminated) implant was left in situ. Among 36 different antibiotic regimens, the bulk of antimicrobial therapy was taken orally. Intravenous agents were applied only during the initial postoperative days. We equally renounced using local (intraosseous) antimicrobial deliveries [13,14] or topical superficial agents [13,14], as they were reserved for recurrent and/or ulcerated foot infections. The median duration of postoperative antibiotic use was 42 days (IQR, 27–84 days) and was determined by infectious diseases physicians with experience in orthopedic infections. The three most frequently used antibiotics were co-amoxiclav, clindamycin, and co-trimoxazole. The median number of surgical debridement procedures for infection was 1 (IQR, 1–2 debridement procedures). The surgical management was supported by the use of casts in 12 (33%) episodes, postoperative wounds with negative-pressure devices [15] in eight (22%) cases, and partial off-loading in all episodes. In terms of treatment outcomes, 29 out of 36 patients (80.56%) achieved full remission and remained in remission after a median active surgical follow-up time of 4.3 years (IQR, 2.6–6.6 y). Seven episodes (19%) failed at the same anatomical localization, but new pathogens were eventually detected. None of them yielded a microbiologically identical relapse with the same pathogen(s) as in the index infection.

### 3.3. C-Reactive Protein Levels Associated to Outcomes

Overall, the median preoperative CRP level was 25 mg/L (IQR, 8–48 mg/L). The postoperative median CRP level on days 6–13 was 8.9 mg/L (IQR, 4–22 mg/L). The median CRP level in weeks 5 to 8 was 28.2 mg/L (IQR, 8–70 mg/L). The median CRP level in weeks 11 to 14 was 37.5 mg/L (IQR, 11–93 mg/L). The median relative drop (ratio) in the CRP level between the presurgical value and the first postoperative level was 75% (IQR, 1–94%). At the end of therapy, five patients (14%) could not normalize their CRP levels to baseline and/or ≤10 mg/L without continuing the antibiotics. Table 1 compares key variables and serum CRP levels between remissions and failures.

**Figure 1:**
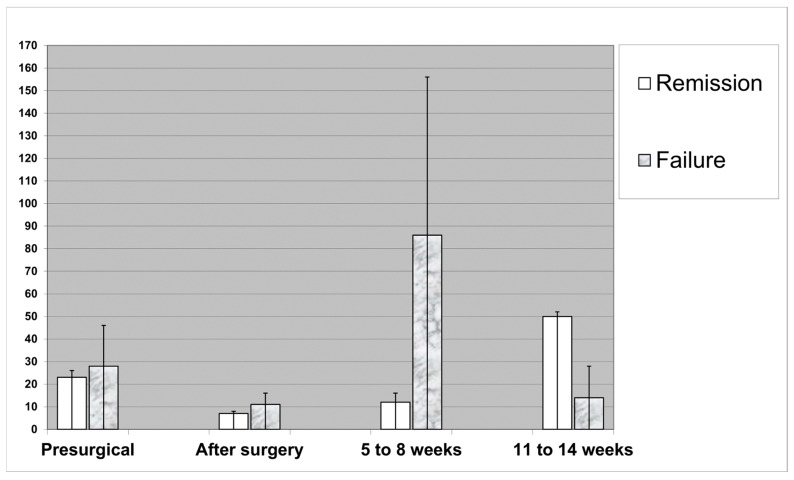
Serum C-reactive protein values on admission, after surgery, and at the end of treatment. Left vertical axis: median CRP values. The thin lines represent the 95% confidence intervals.

The CRP levels showed a very large dispersion and variance, both between the individual patients and over time for a given SSI episode (Figure 1). Although the immediate drop after surgery was parallel between the groups in terms of “remission” and “failure”, the further course became much more aleatory. Equally, the trends in the measured CRP levels (independent of their absolute value) could not predict “failure”. For example, from week 5 to 8, the CRP levels seemed more dispersed among those who failed (with the occurrence of important outliers); the situation was even inversed between weeks 11 and 14 when the local visual aspects “calmed down” (Figure 1). However, as a clinical consequence, the medical records indicate high medical activity after the detection of unsurprisingly high CRP values. For example, these values were contributing to delaying the postsurgical transfer to a rehabilitation center; or postponing the change from a parenteral to an oral antibiotic prescription; or performing unplanned and costly examinations such as urine cultures in asymptomatic patients, unplanned X-rays, or other radiological examinations for the exclusion of hidden abscesses, which might have even eventually prolonged the individual length of the hospital stay.

### 3.4. Multivariate Adjustment

Due to the large case mix, we adjusted with a logistic regression analysis and its outcome of “treatment failure”. In this multivariate analysis, the pre- and postsurgical CRP values oscillated around an odds ratio of 1.0. The 95% confidence intervals were narrow (Table 2), the goodness-of-fit-test was insignificant, and the Receiver under the Curve (ROC) value was 0.83, representing the more-than-adequate accuracy of our final statistical model. These results represent a very limited influence of the iterative CRP on the ultimate fate of therapy.

## 4. Discussion

We cannot display a significant association between routine, singular, and serial serum CRP samplings in the aftermath of surgical debridement for orthopedic foot SSIs. In our study, the absolute CRP control values, their timely trends, or their interpersonal variabilities all failed to predict the future “failure” of treatment of orthopedic foot SSIs. Our results are in line with our own clinical experience and the general orthopedic literature, and is also underlined by experts in orthopedic infectious diseases. The existing literature has almost exclusively investigated the accuracy of serum CRP levels in the diagnosis of a suspected infection, which is beyond the scope of our study. Rarely, serial serum CRP controls have been investigated in orthopedic surgery.

There have been very few prospective studies examining the performance of iterative CRP measures during the follow-up of already infected orthopedic patients. Dupont et al. sampled different serum inflammatory markers after one week, three weeks, and three months of treatment [6]. All values declined after the initiation of antibiotics. The serum CRP values returned to near-normal levels on day 21. However, it remained unclear as to the clinical benefit of routine CRP monitoring (or what would have missed by clinical evaluation alone) [6]. To cite another clinical example, a French study monitored arthroplasties. The authors concluded that a clinical local visual discharge, a fever >38 °C, and local/persistent pain were more informative indicators of postoperative infection complications than the measured evolution of CRP levels [16]. In another British study with 260 infected arthroplasties, the serial sampling of 3732 serum CRP levels turned out to be a very poor predictor of general outcome and, thus, was not recommended by the authors themselves [17]. Of note, their area-under-the-ROC curves for CRP levels predicting a good outcome only ranged from 0.55 to 0.65, performing only slightly better than a coin flip [17]. A German group compared the diagnostic value of serum CRP levels and the serum white blood cell (WBC) count during a two-stage revision surgery of infected hip arthroplasties. The postoperative courses of the mean CRP values were similar between those who remained reinfection-free and those who experienced a re-infection [18]. The authors concluded that the CRP level and WBC count are not helpful to guide decision-making in individual cases [18]. In the elderly DFI population, the quantitative serum CRP level is dynamic and influenced by many inherent co-morbidities such as gout, cancer, rheumatic diseases, thrombosis, statin drugs, Charcot foot arthropathy [19], hematoma, ischemia, dialysis, cirrhosis, trauma, obesity, or being postoperative [9,20]. Furthermore, the CRP peak always lags 2–3 days behind the event with a long recovery time, making its timely interpretation impracticable [7,21].

The CRP level per se is not decisive or predictive enough to change an antibiotic treatment, or to prolong a scheduled therapy principally based on its values. The serum CRP level is probably not better than clinical surgical decisions, e.g., regarding a second look. In contrast, the routine sampling of serum CRP has a high potential to cause unexpected troubles. At a minimum, many clinicians become disturbed by surprisingly high laboratory finding and often refer to additional examinations; e.g., bacterial urine culture in asymptomatic patients, X-rays, or other costly examinations to exclude hidden abscesses (local or remote ones), thrombosis, or embolism. Besides new examinations and the compulsory repetition of CRP sampling, the clinical consequences are diverse. Sometimes, clinicians might delay hospital discharge or transfer to rehabilitation, only for precautional surveillance and hoping to know what happens. Or, they might delay the planned switch from intravenous to oral antibiotic therapy, hoping that parenteral treatment would decrease the control CRP values faster than oral agents [22]. In the worst case, they (empirically) may broaden the antibiotic spectrum, especially in polymicrobial DFIs. In the best case, they may require a consultation with an infectious diseases clinician [23], which the patient would not have had if his/her CRP levels were low. The authors of this article strongly advocate for the abandonment (to de-implement this entirely) of serum CRP measurement during the treatment of infected orthopedic patients for routine purposes.

Our study has a major strength and several limitations. We focused on uneventful postoperative courses after the first debridement procedure for an SSI, without new events that might alter the indications for CRP measurement for new complications such as lung embolism, ischemia (especially in the diabetic foot), or nosocomial urinary tract infections [24]. The major limitation is the retrospective nature of our study, because retrospective trials cannot randomize and are inherently prone to clinical “confounding by indication” in their analyses. A randomized controlled trial, assessing the decisional benefit of routine measurements would link antibiotic cessation for orthopedic patients with the normalization of the serum CRP level. We have not identified any trial in the orthopedic field that has investigated this. In many other fields of infectious diseases, the cessation of systemic antibiotic use after the achievement of CRP level normalization has been debated for decades and research regularly denies its practicability or benefit. For instance, according to the latest trial in Gram-negative bacteremia, the investigators randomized adult hospitalized patients receiving microbiologically efficacious antibiotic(s) between 14 days of antibiotic therapy, 7 days of therapy, or an individualized duration determined by clinical response, with a 75% reduction in peak CRP values [25]. The preliminary results indicate no differences between all three randomization arms. Generally speaking, in orthopedic infections, the duration of antibiotic treatment is determined by expert opinion and few randomized data, and not on a single laboratory parameter.

Secondly, we sampled the serum CRP levels. Our findings cannot be applied to other inflammatory markers such as pro-calcitonin [9]; the erythrocyte sedimentation rate; interleukins 2, 6 or 8; serum leukocyte counts; tumor necrosis factors; monocyte chemotactic proteins; macro-phage inflammatory proteins; neutrophil-to-lymphocyte ratios; procollagen type 1 N pro-peptides; high-resolution CRPs; or genetically altered CRP peptides [26]. These latter markers are very seldomly used in daily clinical practice and all are more expensive to monitor than standard CRP [9].

Lastly, we investigated the pertinence of measuring serial serum CRP levels in infected-foot patients. There are also surgical groups that measure during not-yet-infected orthopedic surgeries (especially arthroplasties [17]), which is a distinct entity. Follow-up CRP measurements during treatment are informative, but complete normalization is not a mandatory requirement for a successful outcome [27,28,29,30]; meanwhile, in turn, a normalized C-reactive protein level does not rule out chronic infections such as periprosthetic joint infections [29,30,31].

## 5. Conclusions

In our single-center composite foot database, and according to two analogue studies [9,10] in the diabetic foot, routine CRP samples, taken at different time points during ongoing therapy for postoperative SSIs in orthopedic foot surgery, failed to predict failures during and after therapy. At the same time, our literature review could not identify any contradicting publications, and international guidelines are not in favor of this practice. We would recommend abandoning this practice, because the unnecessary samples seem to be a waste of money, requiring inutile phlebotomies for patients and nurses, excessive work-ups, delays in patient transfers, and a delayed switch to (more convenient) oral antibiotic medication. It is likely and more logical that the serum CRP levels could be used, together with a through clinical evaluation, in the diagnostic work-up of new-onset inflammation or a complication during the combined surgical and antibiotic treatment of (diabetic) foot SSIs.

## Figures and Tables

**Table 1:** Clinical variables and serum C-reactive protein levels during and after the treatment of foot infections.

	Remission	Failures	*p*
n = 36	n = 29	n = 7	value
Male sex, n = 15	12 (41%)	3 (43%)	0.94
Median age	61 years	53 years	0.58
American Society of Anesthesiologists’ Score 3 points	13 (45%)	4 (57%)	0.56
Charcot neuroarthropathy, n = 5	2 (7%)	3 (43%)	0.01
Mainly bone infection, n = 24 (more than soft tissues)	19 (66%)	5 (71%)	0.77
Infected osteosynthesis material in situ, n = 7	6 (21%)	1 (14%)	0.70
Median number of surgical debridement procedures	1 intervention	1 intervention	0.15
Median duration of postoperative antibiotic therapy	42 days	47 days	0.54
Median CRP level on admission	23 mg/L	28 mg/L	0.55
Median CRP level after surgery	7 mg/L	11 mg/L	0.22
Median drop inn CRP level (ratio second/admission values)	25%	26%	0.52
Median CRP level at end of therapy (11–14 weeks)	49 mg/L	14 mg/L	0.38

**Table 2:** Multivariate associations with the outcome “remission” after the therapy for foot infections (Unconditional logistic regression analysis; results expressed as odds ratios with 95% confidence intervals).

n = 36	Multivariate Results
Age (continuous variable)	1.1, 0.97–1.23
Diabetes mellitus	1.0 (omitted from final model)
Osteosynthesis material	1.0 (omitted from final model)
Concomitant Charcot foot pathology	0.1, 0.01–5.2
Serum CRP level on admission (continuous variable)	1.0, 0.97–1.02
Serum CRP level immediately postoperatively	1.0, 0.94–1.05

## Data Availability

We may provide anonymized key variables upon receipt of a justified scientific request to the last author.
